# Determinants of diagnostic delay in thyroid storm: a 12-year single-center cohort study

**DOI:** 10.1210/jendso/bvag088

**Published:** 2026-04-06

**Authors:** Tetsuya Kawahara, Mikio Toda, Maiko Kanagawa, Nagahiro Toyama, Chie Kawahara, Tetsuya Inazu

**Affiliations:** Division of Endocrinology and Metabolism, Shinkomonji Hospital, Kitakyushu, Fukuoka 800-0057, Japan; Department of Internal Medicine, University of Occupational and Environmental Health, Kitakyushu, Fukuoka 525-0058, Japan; Division of Endocrinology and Metabolism, Shinkomonji Hospital, Kitakyushu, Fukuoka 800-0057, Japan; Department of Internal Medicine, Shinkomonji Hospital, Kitakyushu, Fukuoka 800-0057, Japan; Department of Internal Medicine, Shinkomonji Hospital, Kitakyushu, Fukuoka 800-0057, Japan; Department of Internal Medicine, University of Occupational and Environmental Health, Kitakyushu, Fukuoka 525-0058, Japan; Department of Pharmacy, College of Pharmaceutical Science, Ritsumeikan University, Kusatsu, Shiga 525-8577, Japan

**Keywords:** thyroid storm, diagnostic delay, time to diagnosis, emergency care, thyrotoxicosis

## Abstract

**Objective:**

Thyroid storm is a life-threatening endocrine emergency. Although predictors of mortality have been described, determinants of diagnostic delay remain unclear. We aimed to identify factors associated with diagnostic delay in thyroid storm and its relationship with severe clinical outcomes.

**Methods:**

We conducted a retrospective cohort study of adults admitted with thyroid storm at a tertiary-care hospital over 12 years. The primary endpoint was time from symptom onset to diagnosis (days). Factors associated with time to diagnosis were evaluated using multivariable linear regression adjusting for predefined covariates, including age, initial onset vs recurrence, admitting department, presenting symptoms, and cardiac rhythm abnormalities. Secondary analyses examined delayed diagnosis (≥7 days) using multivariable logistic regression. Additional analyses separated diagnostic delay into patient-related and in-hospital components and evaluated admission pathways and referral status.

**Results:**

Ninety-one patients were included. Median time to diagnosis was 5 days (interquartile range 3.5-7). Older age (1.08 days per 10-year increase; *P* < .001), initial onset (4.10 days; *P* < .001), and admission to non-emergency departments (2.76 days; *P* = .004) were independently associated with longer diagnostic delay, whereas symptom patterns and thyroid hormone levels were not. Patient-related delay did not differ by admission pathway, but in-hospital delay was longer among non-emergency admissions. Delayed diagnosis was associated with increased need for intensive care and organ support.

**Conclusion:**

Diagnostic delay in thyroid storm appears driven mainly by clinical pathways rather than symptom patterns or biochemical severity. Earlier consideration of thyroid storm and prompt thyroid testing—particularly at initial onset and in non-emergency settings—may improve outcomes.

The incidence of thyroid storm in the general population is estimated to be 0.2-1.0 cases per 100 000 persons per year, making it a rare condition [1-3]. However, when it occurs, thyroid crisis is characterized by severe thyrotoxicosis accompanied by acute multiorgan dysfunction and constitutes a life-threatening endocrine emergency [4, 5]. Despite advances in intensive care and guideline-based management, mortality remains substantial [6-8], particularly when diagnosis and treatment are delayed [9]. Previous studies have identified several predictors of mortality in thyroid storm, including advanced age, heart failure, disseminated intravascular coagulation, and central nervous system dysfunction at admission [4, 7, 10, 11]. In contrast, the role of diagnostic delay itself has received far less attention.

Clinical recognition of thyroid storm is often challenging [12-14]. Presenting features may be nonspecific and overlap with more common conditions such as infection, heart failure, or gastrointestinal disease. This is especially true in older adults, in whom so-called “apathetic” presentations lacking overt adrenergic features have been described [1, 15]. In addition, thyroid storm may occur as an initial onset of thyrotoxicosis [16], further reducing clinical suspicion. As a result, diagnosis may be deferred until patients develop overt multiorgan failure, at which point prognosis worsens despite appropriate treatment.

To date, few studies have systematically examined time from symptom onset to diagnosis in thyroid storm, and the determinants of diagnostic delay remain poorly defined. Moreover, it is unclear whether diagnostic delay reflects patient-related factors, symptom patterns, or healthcare system pathways, such as the initial admitting department. Understanding these factors is critical, as diagnostic delay represents a potentially modifiable contributor to adverse outcomes.

In this study, therefore, we conducted a 12-year retrospective cohort analysis of patients admitted with thyroid storm at a tertiary-care center. Our objectives were to characterize time to diagnosis as a continuous clinical endpoint, identify patient- and system-level factors associated with diagnostic delay, and examine the relationship between diagnostic delay and severe clinical outcomes.

## Materials and methods

### Study design and setting

This is a retrospective cohort study conducted at a single tertiary-care hospital. We reviewed all consecutive adult patients admitted with a diagnosis of thyroid storm over 12 years, from January 2014 to January 2026.

Patients were eligible for inclusion if they were aged ≥18 years and met diagnostic criteria for thyroid storm during hospitalization. Diagnosis was based on the clinical judgment of the attending endocrinologists, supported by established diagnostic criteria, as described below [3, 5], that were in use at the time of admission or during hospitalization.

Diagnostic criteria for thyroid storm (Table S1) [17]

#### TS1 (definite thyroid storm)

The patient has biochemically confirmed thyrotoxicosis (elevated serum free triiodothyronine [FT3] and/or free thyroxine [FT4] levels with suppressed serum thyroid-stimulating hormone [TSH] levels) and meets one of the following criteria:

1. Central nervous system manifestations (eg, agitation, delirium, or coma), and at least one of the following:
  Fever (≥38 °C)
  Tachycardia (heart rate ≥ 130 beats per minute)
  Congestive heart failure
  Gastrointestinal and/or hepatic manifestations (eg, diarrhea, vomiting, or jaundice)
2. Three or more of the following features
  Fever (≥38 °C)
  Tachycardia (heart rate ≥ 130 beats per minute)
  Congestive heart failure
  Gastrointestinal and/or hepatic manifestations

#### TS2 (suspected thyroid storm)

The patient has thyrotoxicosis and meets one of the following criteria:

1. A combination of 2 major features:
  Fever (≥38 °C)
  Tachycardia (heart rate ≥ 130 beats per minute)
  Congestive heart failure
  Gastrointestinal and/or hepatic manifestations
2. Clinical features consistent with TS1 in the absence of available thyroid function tests
  A documented history of thyroid disease
  Characteristic physical findings, such as exophthalmos and/or goiter

### Data collection

We stored the ID numbers of patients diagnosed with thyroid storm in advance. Clinical data were extracted from electronic medical records using a standardized data collection form. Collected variables included demographic characteristics, presenting symptoms, vital signs at admission, laboratory data, cardiac rhythm, and clinical course during hospitalization. Presenting symptoms were categorized into gastrointestinal symptoms (including nausea, vomiting, diarrhea, and abdominal pain), neuropsychiatric symptoms, and fever elevation. Cardiac rhythm abnormalities were defined as sinus tachycardia or tachyarrhythmia (including atrial fibrillation, atrial flutter, or supraventricular tachycardia) with a heart rate ≥130 beats per minute on the admission electrocardiogram.

Thyroid function tests, including serum TSH, FT3, and FT4, were recorded at the time of diagnosis. In addition, thyroid antibodies were measured in all patients.

### Definition of key variables

#### Time to diagnosis: primary endpoint

Time to diagnosis was defined as the number of days from patient-reported symptom onset to the date on which thyroid storm was diagnosed and specific treatment for thyroid storm was initiated. Conceptually, this interval includes both patient-related delay and in-hospital delay. When symptom onset was documented as a range, the earliest reported date was used.

#### Diagnostic delay

For secondary and sensitivity analyses, diagnostic delay was defined using 2 predefined thresholds:

- ≥7 days, corresponding to the upper quartile of the distribution (primary definition of delayed diagnosis), and
- ≥median (5 days).

#### Patient delay and in-hospital diagnostic delay

Patient delay was defined as the interval from patient-reported symptom onset to first medical contact, including emergency department visits, outpatient (non-emergency department) consultations, or referrals. In-hospital diagnostic delay was defined as the interval from hospital admission to the diagnosis of thyroid storm and initiation of storm-specific treatment. Because thyroid function test is routinely available within approximately one hour of ordering in our institution and treatment is initiated immediately upon diagnostic confirmation, admission-to-diagnosis time was considered operationally equivalent to admission-to-thyroid function test ordering time.

#### Initial onset

Initial presentation was defined as thyroid storm occurring in patients without a prior documented diagnosis of hyperthyroidism or thyrotoxicosis. Patients with a previously documented diagnosis of hyperthyroidism or thyrotoxicosis were classified as having known thyroid disease.

#### Admitting department

The admitting department was classified based on the service to which the patient was first admitted. For primary analyses, this variable was dichotomized as emergency department vs non-emergency departments, such as general internal medicine, gastroenterology, psychology, and cardiology.

#### Admission pathway and referral status

Admission pathway was categorized as emergency department vs non-emergency departments. Referral from another hospital was defined as patients who were initially evaluated and admitted at another institution before being transferred to our center for definitive management. This variable was included to distinguish inter-hospital referral from within-hospital admission pathways.

### Clinical outcomes

Severe clinical outcomes assessed during hospitalization included admission to the intensive care unit, use of vasopressors, requirement for mechanical ventilation, and development of multiorgan failure. In-hospital mortality was recorded but analyzed descriptively due to the small number of events.

The study protocol was approved by the institutional review board of Shinkomonji Hospital. Given the retrospective nature of the study and use of anonymized data, the requirement for informed consent was waived.

### Statistical analysis

Continuous variables are presented as mean ± standard deviation or median with interquartile range, as appropriate, and categorical variables as counts and percentages. Comparisons between groups were performed using the Student's *t*-test or Mann–Whitney *U* test for continuous variables and the χ^2^ test or Fisher's exact test for categorical variables, as appropriate. The primary endpoint was time from symptom onset to diagnosis of thyroid storm, expressed as a continuous variable (days). Factors associated with time to diagnosis were evaluated using multivariable linear regression.

For secondary and sensitivity analyses, diagnostic delay was additionally dichotomized using 2 predefined thresholds: ≥7 days (upper quartile, primary definition of delayed diagnosis) and ≥ the median (5 days). Associations between dichotomized diagnostic delay and clinical outcomes were examined using multivariable logistic regression models. Covariates selected *a priori* based on clinical relevance and prior literature were entered simultaneously into the multivariable linear regression model. They included age, initial onset vs recurrence, admitting department (emergency vs non-emergency departments), gastrointestinal symptoms, fever elevation, neuropsychiatric symptoms, and cardiac rhythm abnormalities. Model assumptions were assessed using residual diagnostics, and no major violations were identified.

Differences in patient delay and in-hospital diagnostic delay between emergency and non-emergency department admissions were compared using the Mann–Whitney *U* test. Factors associated with in-hospital diagnostic delay were examined using multivariable linear regression, adjusting for age, initial onset vs recurrence, admission pathway, referral from another hospital, presenting symptoms, and cardiac rhythm abnormalities. Sensitivity analyses were performed using robust regression with a Huber M-estimator to account for skewed distributions and potential outlier influence.

Because the number of in-hospital deaths was small (*n* = 3), mortality was analyzed descriptively and not used as a primary outcome. All statistical tests were 2-sided, and a *P*-value <.05 was considered statistically significant. Analyses were performed using R software (version 4.5.1). Because this was a retrospective analysis of all consecutive cases over the 12-year study period, no a priori sample size calculation was performed.

## Results

### Patient characteristics

The 92 patients who were ultimately diagnosed with TA during the 12-year study period were identified. One patient was excluded because of missing data for symptom onset, leaving 91 patients for the primary analysis.

**Figure 1:**
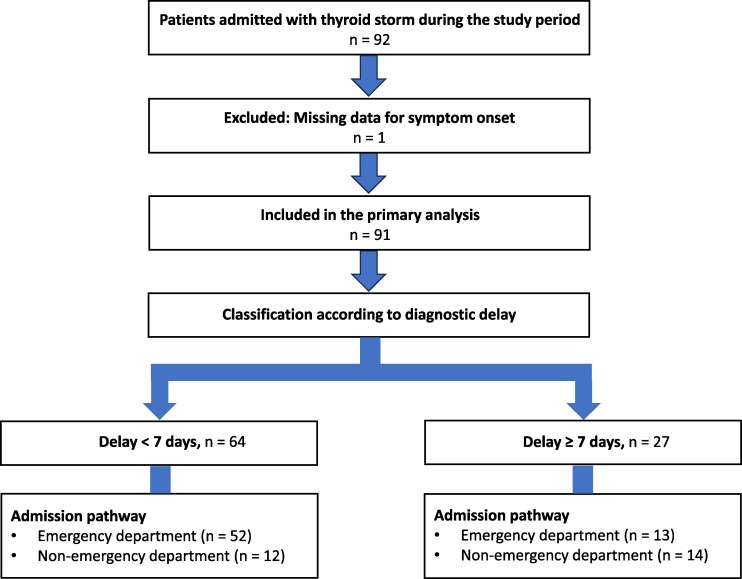
Patient flow diagram of the study population. Of 92 patients admitted with thyroid storm during the study period, one patient was excluded because of missing data on symptom onset. The remaining 91 patients were included in the primary analysis and classified according to diagnostic delay (<7 days vs ≥7 days). Admission pathways included emergency department and non-emergency department presentations.

The study population flow diagram is shown in Fig. 1. Baseline characteristics of the study population are summarized in Table 1. The median time to diagnosis was 5 days (interquartile range, 3.5–7days), with 27 patients (29.7%) experiencing a diagnostic delay of ≥7 days. Baseline characteristics stratified by diagnostic delay are shown in Table 2. Patients with diagnostic delay ≥7 days were older, more likely to present with initial onset, and more frequently admitted to non-emergency departments. Graves' disease was the predominant etiology (86 cases, 94.5%), whereas non-Graves etiologies were uncommon (toxic multinodular goiter, *n* = 1; toxic adenoma, *n* = 1; destructive thyroiditis, *n* = 3). Because of the small number of non-Graves cases, disease-specific comparisons were not feasible. When grouped as Graves vs non-Graves etiologies (86 vs 5 cases), no association with diagnostic delay was observed (Fisher's exact test, *P* = .65).

**Table 1:** Baseline characteristics

Characteristic	Overall (*n* = 91)
**Age, years**	47.5 (10.0)
**Sex male, *n* (%)**	34 (37.4)
**Initial onset, *n* (%)**	32 (35.2)
**Heart rate, beats/min**	94.0 (11.8)
**Systolic blood pressure (mmHg)**	117.1 (13.2)
**Cardiac rhythm abnormalities** *^a^* **, *n* (%)**	27 (29.5)
**Fever elevation** *^b^* **, *n* (%)**	66 (72.5)
**Neuropsychiatric symptom, *n* (%)**	38 (41.8)
**Gastrointestinal symptom, *n* (%)**	42 (46.2)
**Weight loss** *^c^* **, *n* (%)**	69 (75.8)
**Admission to non-emergency departments, *n* (%)**	26 (28.6)
**Thyroid function**	
** TSH (μIU/mL) [0.61–4.23]**	0.01 (0.01–0.01)
** FT3 (pg/mL) [2.3–4.0]**	30.0 (24.0–30.0)
** FT4 (ng/dL) [0.9–1.7]**	8.00 (6.90–8.00)
**Time to diagnosis, days**	5 (3.5–7.0)
**Delayed diagnosis ≥7 days, *n* (%)**	27 (29.7)

Data are presented as mean (SD), median (interquartile range), or number (percentage), as appropriate. [ ] indicates the normal range.

^*a*^Cardiac rhythm abnormalities were defined as sinus tachycardia or tachyarrhythmia (including atrial fibrillation, atrial flutter, or supraventricular tachycardia) with a heart rate ≥130 beats per minute on the admission electrocardiogram.

^*b*^Fever elevation was defined as a body temperature ≥ 38.0 °C.

^*c*^Weight loss was defined as a ≥5% reduction in body weight within one month or an equivalent reduction over a shorter period.

**Table 2:** Baseline characteristics stratified by diagnostic delay (≥7 days vs <7 days)

Characteristic	Overall (*n* = 91)	<7 days (*n* = 64)	≥7 days (*n* = 27)	*P* value
**Age, years**	47.5 (10.0)	42.1 (9.4)	60.3 (8.7)	<.001
**Sex male, *n* (%)**	34 (37.4)	25 (39.1)	9 (33.3)	.60
**Initial onset, *n* (%)**	32 (35.2)	13 (20.3)	19 (70.4)	<.001
**Heart rate, beats/min**	94.0 (11.8)	95.1 (11.5)	91.6 (12.2)	.18
**Systolic blood pressure (mmHg)**	117.1 (13.2)	119.1 (12.0)	114.9 (13.0)	.24
**Cardiac rhythm abnormalities** *^a^* **, *n* (%)**	27 (29.5)	18 (28.1)	9 (33.3)	.62
**Fever elevation** *^b^* **, *n* (%)**	66 (72.5)	47 (73.4)	19 (70.4)	.77
**Neuropsychiatric symptom, *n* (%)**	38 (41.8)	26 (40.6)	12 (44.4)	.74
**Gastrointestinal symptom, *n* (%)**	42 (46.2)	27 (42.2)	15 (55.6)	.25
**Weight loss** *^c^* **, *n* (%)**	69 (75.8)	47 (73.4)	22 (81.5)	.59
**Admission to non-emergency departments, *n* (%)**	26 (28.6)	12 (18.8)	14 (51.9)	.002
**Thyroid function**				
** TSH (μIU/mL) [0.61-4.23]**	0.01 (0.01-0.01)	0.01 (0.01-0.01)	0.01 (0.01-0.01)	.91
** FT3 (pg/mL) [2.3-4.0]**	30.0 (24.0-30.0)	29.5 (24.0-30.0)	30.0 (24.0-30.0)	.68
** FT4 (ng/dL) [0.9-1.7]**	8.00 (6.90-8.00)	7.9 (6.8-8.0)	8.0 (7.0-8.0)	.71

Data are presented as mean (SD), median (interquartile range), or number (percentage), as appropriate. [ ] indicates the normal range.

*P*-values were calculated using t test or Mann–Whitney *U* test, and χ^2^ or Fisher's exact test, as appropriate.

^*a*^Cardiac rhythm abnormalities were defined as sinus tachycardia or tachyarrhythmia (including atrial fibrillation, atrial flutter, or supraventricular tachycardia) with a heart rate ≥130 beats per minute on the admission electrocardiogram.

^*b*^Fever elevation was defined as a body temperature ≥38.0 °C.

^*c*^Weight loss was defined as a ≥5% reduction in body weight within one month or an equivalent reduction over a shorter period.

### Primary endpoint: time to diagnosis (continuous outcome)

In multivariable linear regression analysis, older age, initial onset, and admission to non-emergency departments were independently associated with a longer time to diagnosis (Table 3). Specifically, each 10-year increase in age was associated with an additional 1.08 days to diagnosis (95% CI, 0.52-1.64; *P* < .001). Patients experiencing an initial onset had a diagnosis delayed by an average of 4.10 days compared with those with recurrent disease (95% CI, 3.09-5.10; *P* < .001). Admission to non-emergency departments was associated with an additional 2.76 days to diagnosis compared with admission through the emergency department (95% CI, 0.88-4.64; *P* = .004). In contrast, gastrointestinal symptoms, fever elevation, neuropsychiatric symptoms, and atrial fibrillation were not independently associated with time to diagnosis after adjustment.

**Table 3:** Factors associated with time to diagnosis (days)

Variable	β coefficient (days)	95% CI	*P*-value
**Age (per 10-year increase)**	1.08	0.52-1.64	<.001
**Initial onset (vs recurrence)**	4.10	3.09-5.10	<.001
**Admission to non-emergency departments (vs emergency)**	2.76	0.88-4.64	.004
**Gastrointestinal symptom**	0.42	−0.68-1.53	.44
**Fever elevation** *^a^*	−0.51	−1.54-0.53	.33
**Neuropsychiatric symptom**	0.07	−1.15-1.30	.91
**Cardiac rhythm abnormalities** *^b^*	−0.02	−1.28-1.34	.98

Model fit: *R*^2^ = 0.647, adjusted *R*^2^ = 0.621, overall model *P* < .001.

^*a*^Fever elevation was defined as a body temperature ≥38.0 °C.

^*b*^Cardiac rhythm abnormalities were defined as sinus tachycardia or tachyarrhythmia (including atrial fibrillation, atrial flutter, or supraventricular tachycardia) with a heart rate ≥130 beats per minute on the admission electrocardiogram.

### Secondary analyses: factors associated with dichotomized diagnostic delay

Diagnostic delay was dichotomized using 2 predefined thresholds: ≥7 days (upper quartile) and ≥ median (5 days) (Table 4). Using the ≥7-day definition, delayed diagnosis was independently associated with older age (OR 1.34 per 10-year increase; 95% CI, 1.18-1.52; *P* < .001), initial onset (OR 4.82; 95% CI, 2.12-10.95; *P* < .001), and admission to non-emergency services (OR 3.21; 95% CI, 1.18-8.73; *P* = .022). No symptom was independently associated with delayed diagnosis. Results were consistent when diagnostic delay was defined as ≥ median (5 days), with all 3 factors remaining statistically significant and in the same direction.

**Table 4.** Factors associated with delayed diagnosis of thyroid storm

Variable	Delayed diagnosis (≥7 days) OR (95% CI)	*P*-value	Delayed diagnosis (≥5days) OR (95% CI)	*P*-value
**Age (per 10-year increase)**	1.34 (1.18-1.52)	<.001	1.29 (1.16-1.44)	< .001
**Initial onset (vs recurrence)**	4.82 (2.12-10.95)	<.001	3.67 (1.78-7.56)	<.001
**Admission to non-emergency departments (vs emergency)**	3.21 (1.18-8.73)	.022	2.47 (1.08-5.63)	.032
**Gastrointestinal symptom**	1.21 (0.55-2.67)	.64	1.14 (0.57-2.30)	.71
**Fever elevation** *^a^*	0.92 (0.43-1.96)	.83	0.98 (0.50-1.93)	.96
**Neuropsychiatric symptom**	1.03 (0.44-2.40)	.95	1.01 (0.46-2.24)	.98
**Cardiac rhythm abnormalities** *^b^*	1.00 (0.41-2.46)	.99	1.05 (0.46-2.39)	.91

Odds ratios were estimated using multivariable logistic regression adjusting for all listed variables.

Delayed diagnosis was defined a priori as ≥7 days (upper quartile) and reassessed using the median (≥5 days) for sensitivity analysis.

^*a*^Fever elevation was defined as a body temperature ≥38.0 °C.

^*b*^Cardiac rhythm abnormalities were defined as sinus tachycardia or tachyarrhythmia (including atrial fibrillation, atrial flutter, or supraventricular tachycardia) with a heart rate ≥130 beats per minute on the admission electrocardiogram.

### Conceptual framework of diagnostic delay in thyroid storm

**Figure 2:**
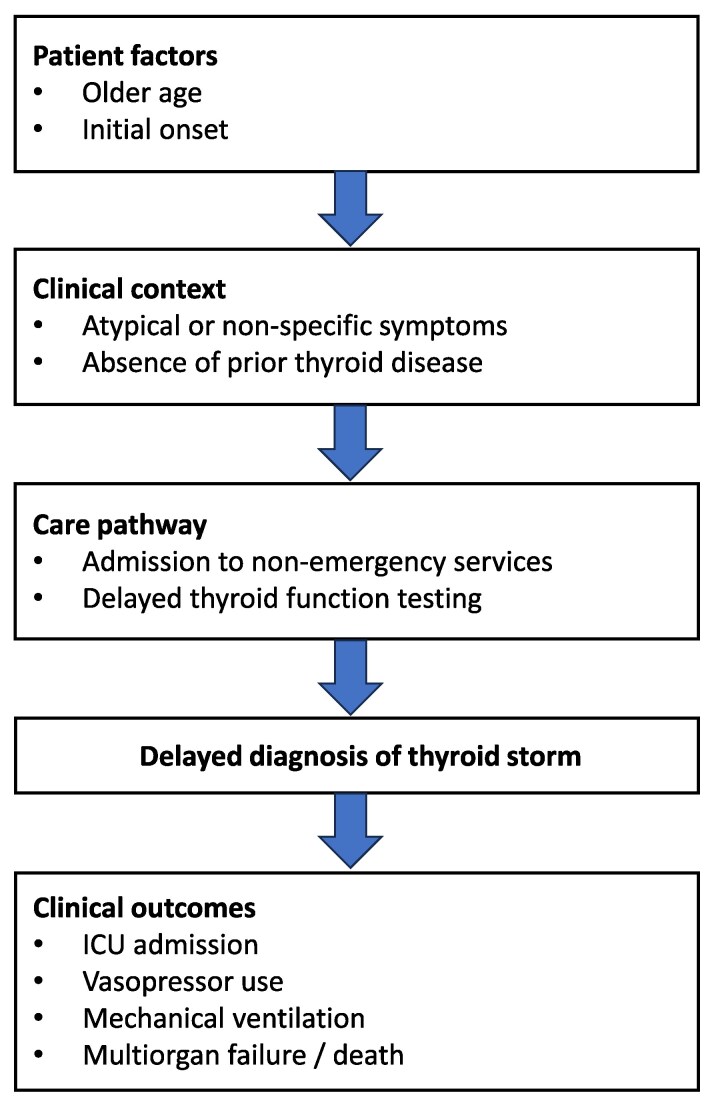
Conceptual framework of diagnostic delay in thyroid storm. Conceptual framework illustrating the relationships between patient characteristics, clinical context, and care pathways contributing to diagnostic delay in thyroid storm. Older age and initial onset are associated with atypical or non-specific symptoms and absence of prior thyroid disease, which may lead to admission to non-emergency services and delayed thyroid function testing. Diagnostic delay is in turn associated with severe clinical outcomes, including intensive care unit admission, vasopressor use, mechanical ventilation, multiorgan failure, and death.

The conceptual framework linking patient characteristics, care pathways, and diagnostic delay in thyroid storm is shown in Fig. 2. Older age and initial onset were associated with non-emergency admission pathways and delayed thyroid function testing, which in turn were associated with delayed diagnosis and severe clinical outcomes.

### Decomposition of diagnostic delay

When diagnostic delay was decomposed into patient-related and in-hospital components, patient delay did not differ significantly between emergency and non-emergency department admissions (median 2 days in both groups; Table S2) [17]. In contrast, in-hospital diagnostic delay was significantly longer among patients admitted to non-emergency departments, with a median delay of 4 days compared with 2 days among emergency department admissions (*P* < .001; Table S2) [17].

### Determinants of in-hospital diagnostic delay

In multivariable linear regression analysis, admission to non-emergency departments and initial onset of thyroid storm were independently associated with longer in-hospital diagnostic delay (Table S3-A) [17]. Among patients admitted to non-emergency departments (*n* = 26), the initial admitting services included general internal medicine (*n* = 10), gastroenterology (*n* = 8), cardiology (*n* = 2), and psychiatry (*n* = 2). After adjustment for admission pathway and referral from another hospital, the association between age and in-hospital diagnostic delay attenuated and was no longer statistically significant. Referral from another hospital was not independently associated with admission-to-diagnosis time. These findings were consistent in sensitivity analysis using robust regression (Table S3B) [17]. The distribution of in-hospital diagnostic delay by admission pathway is shown in Fig. S1 [17], illustrating a right-skewed distribution with more extreme delays among non-emergency admissions.

### Diagnostic delay and severe clinical outcomes

Delayed diagnosis (≥7 days) was associated with significantly increased odds of ICU admission, vasopressor use, mechanical ventilation, and multiorgan failure after adjustment for age, initial onset, and admitting department (Table 5). Similar associations were observed using the ≥ median (5 days) definition. Because the number of in-hospital deaths was small (*n* = 3), mortality was analyzed descriptively. All fatal cases occurred in the delayed-diagnosis group.

**Table 5:** Association between diagnostic delay and severe clinical outcomes

Outcome	Delayed diagnosis (≥7days) OR (95% CI)	*P*-value	Delayed diagnosis (≥5days) OR (95% CI)	*P*-value
**ICU admission**	2.61 (1.16-5.86)	.02	2.14 (1.06-4.31)	.03
**Vasopressor use**	2.48 (1.10-5.60)	.03	2.01 (1.01-4.01)	.047
**Mechanical ventilation**	2.73 (1.12-6.67)	.03	2.22 (1.01-4.86)	.048
**Multiorgan failure**	3.08 (1.21-7.82)	.02	2.45 (1.09-5.51)	.03
**In-hospital mortality** *^a^*	Descriptive	**—**	Descriptive	**—**

Odds ratios were estimated using multivariable logistic regression adjusting for age, initial onset, and admitting department.

Abbreviation: ICU, intensive care unit.

^*a*^In-hospital mortality was analyzed descriptively because of the small number of events (*n* = 3); all fatal cases occurred in the delayed-diagnosis group.

## Discussion

This 12-year single-center cohort study demonstrates that diagnostic delay in thyroid storm is associated with patient background and, more importantly, clinical pathways rather than presenting symptom patterns or biochemical severity. Older age, initial onset, and admission to non-emergency departments were associated with longer time to diagnosis, whereas gastrointestinal symptoms, neuropsychiatric manifestations, fever, cardiac rhythm abnormalities, and thyroid hormone levels were not independently predictive. These findings indicate that delayed recognition of thyroid storm reflects failures in diagnostic consideration within specific clinical contexts rather than uniformly delayed care across all patients.

To our knowledge, no prior study has systematically evaluated time to diagnosis as a continuous endpoint and examined its association with severe in-hospital outcomes in thyroid storm. By focusing on diagnostic delay rather than mortality predictors alone, our findings shift attention from disease severity to potentially modifiable clinical pathways.

By decomposing time to diagnosis into patient-related and in-hospital components, our supplementary analyses provide further insight into the mechanisms underlying diagnostic delay. Patient delay did not differ by admission pathway, whereas in-hospital diagnostic delay was significantly prolonged among patients admitted to non-emergency departments. Moreover, the right-skewed distribution of in-hospital diagnostic delay in non-emergency admissions indicates that although many patients were diagnosed promptly, a subset experienced markedly prolonged delays. This pattern suggests that diagnostic delay arises from missed or delayed consideration of thyroid storm in specific settings rather than from generalized inefficiency of care.

Although older age was strongly associated with overall diagnostic delay from symptom onset to diagnosis, this association attenuated when analyses focused specifically on in-hospital diagnostic delay and accounted for admission pathway and referral status. This finding suggests that age itself is not the primary driver of delayed diagnosis but rather acts as a proxy for differences in clinical context, including atypical presentations and admission to non-emergency departments. These results are consistent with prior descriptions of so-called apathetic thyroid storm in older adults [1, 15], in which classical adrenergic features may be attenuated, reducing diagnostic suspicion.

Initial onset was the strongest determinant of diagnostic delay. Patients with thyroid storm at initial onset were frequently misclassified as having other conditions, leading to delayed consideration of the diagnosis. Previous studies have reported that approximately half of patients hospitalized with thyroid storm presented with the condition at initial onset [16] and that 71.4% were not diagnosed on the day of admission [13]. Our findings extend these observations by demonstrating that such delays arise predominantly from in-hospital diagnostic processes rather than delayed healthcare-seeking behavior. In addition, our findings underscore that the absence of a known history of thyroid disease should not lower clinical suspicion for thyroid storm.

Admission pathway also had a substantial impact on diagnostic delay. Admission to non-emergency departments was independently associated with prolonged in-hospital diagnostic delay, even after adjustment for referral from another hospital. This suggests that thyroid storm may be underrecognized in non-emergency settings, where thyroid function testing and endocrinology consultation may be deferred. Given that thyrotoxicosis and hyperthyroidism are commonly encountered in primary care [18, 19], thyroid storm—although rare—should be recognized as a life-threatening condition that can occur across a wide range of clinical settings.

Notably, although diagnostic delay was more frequent among older patients on average, the longest delay in our cohort occurred in a young adult. Regarding the proportion of young adults in observational studies of patients with TS, a Japanese study (*n* = 1324) reported a mean age of 47 ± 18 years, with 58 (4.4%) patients aged <20 years and 443 (33.5%) aged 20–39 years [4]. A German study (*n* = 1690) reported a mean age of 60.1 ± 18.6 years, with 19 (1.1%) patients aged <18 years and 134 (7.9%) aged 18–30 years [1]. These findings suggest that although the age distribution of TS varies across regions and populations, the risk of TS is not negligible in young adults. Our observation further indicates that delayed recognition is not confined to older populations and may occur across all age groups, particularly in cases with atypical presentations or non-emergency admission pathways. These findings support the need for age-independent clinical vigilance.

Several limitations should be acknowledged. This was a single-center retrospective study, which limits generalizability to healthcare systems with different referral structures or diagnostic workflows. Symptom onset was determined partly based on patient-reported history and referral documentation, which may introduce recall bias; however, the earliest documented onset was consistently used to mitigate this limitation. Despite representing one of the larger single-center cohorts of thyroid storm, the number of in-hospital deaths was small, precluding robust mortality modeling. Residual confounding related to unmeasured system-level factors, such as access to endocrinology consultation or institutional testing protocols, cannot be excluded. Finally, although diagnostic criteria and clinical practice may have evolved over the study period, all cases were diagnosed by endocrinology specialists in accordance with the Japan Thyroid Association Guidelines [3, 5], ensuring internal consistency.

## Conclusion

In summary, diagnostic delay in thyroid storm arises predominantly from clinical context and admission pathways rather than from symptom patterns or biochemical severity. Earlier consideration of thyroid storm and timely thyroid function testing—particularly at initial presentation and in non-emergency departments—represents practical, potentially modifiable strategies to reduce diagnostic delay and improve outcomes. To validate the external validity of these findings, we are planning a future multicenter clinical study.

## Data Availability

Some or all datasets generated during and/or analyzed during the current study are not publicly available but are available from the corresponding author on reasonable request.

## Abbreviations

FT3: free triiodothyronine
FT4: free thyroxine
ICU: intensive care unit
TSH: thyroid stimulating hormone
